# EpiRegio: analysis and retrieval of regulatory elements linked to genes

**DOI:** 10.1093/nar/gkaa382

**Published:** 2020-05-27

**Authors:** Nina Baumgarten, Dennis Hecker, Sivarajan Karunanithi, Florian Schmidt, Markus List, Marcel H Schulz

**Affiliations:** Institute for Cardiovascular Regeneration, Goethe University Hospital, 60590 Frankfurt am Main, Germany; Cardio-Pulmonary Institute, Goethe University Hospital, 60590 Frankfurt am Main, Germany; German Center for Cardiovascular Research, Partner site Rhein-Main, 60590 Frankfurt am Main, Germany; Cluster of Excellence, Multimodal Computing and Interaction, Saarland Informatics Campus, 66123 Saarbrücken, Germany; Institute for Cardiovascular Regeneration, Goethe University Hospital, 60590 Frankfurt am Main, Germany; Cardio-Pulmonary Institute, Goethe University Hospital, 60590 Frankfurt am Main, Germany; German Center for Cardiovascular Research, Partner site Rhein-Main, 60590 Frankfurt am Main, Germany; Institute for Cardiovascular Regeneration, Goethe University Hospital, 60590 Frankfurt am Main, Germany; Cardio-Pulmonary Institute, Goethe University Hospital, 60590 Frankfurt am Main, Germany; German Center for Cardiovascular Research, Partner site Rhein-Main, 60590 Frankfurt am Main, Germany; German Center for Cardiovascular Research, Partner site Rhein-Main, 60590 Frankfurt am Main, Germany; Cluster of Excellence, Multimodal Computing and Interaction, Saarland Informatics Campus, 66123 Saarbrücken, Germany; Genome Institute of Singapore, 60 Biopolis Street, Genome, 02-01, 138672, Singapore; Big Data in BioMedicine Group, Chair of Experimental Bioinformatics, TUM School of Life Sciences, Technical University of Munich, Maximus-von-Imhof-Forum 3, 85354 Freising, Germany; Institute for Cardiovascular Regeneration, Goethe University Hospital, 60590 Frankfurt am Main, Germany; Cardio-Pulmonary Institute, Goethe University Hospital, 60590 Frankfurt am Main, Germany; German Center for Cardiovascular Research, Partner site Rhein-Main, 60590 Frankfurt am Main, Germany; Cluster of Excellence, Multimodal Computing and Interaction, Saarland Informatics Campus, 66123 Saarbrücken, Germany

## Abstract

A current challenge in genomics is to interpret non-coding regions and their role in transcriptional regulation of possibly distant target genes. Genome-wide association studies show that a large part of genomic variants are found in those non-coding regions, but their mechanisms of gene regulation are often unknown. An additional challenge is to reliably identify the target genes of the regulatory regions, which is an essential step in understanding their impact on gene expression. Here we present the EpiRegio web server, a resource of regulatory elements (REMs). REMs are genomic regions that exhibit variations in their chromatin accessibility profile associated with changes in expression of their target genes. EpiRegio incorporates both epigenomic and gene expression data for various human primary cell types and tissues, providing an integrated view of REMs in the genome. Our web server allows the analysis of genes and their associated REMs, including the REM’s activity and its estimated cell type-specific contribution to its target gene’s expression. Further, it is possible to explore genomic regions for their regulatory potential, investigate overlapping REMs and by that the dissection of regions of large epigenomic complexity. EpiRegio allows programmatic access through a REST API and is freely available at https://epiregio.de/.

## INTRODUCTION

Research on gene regulation has considerably grown during the last years and is continuously expanding our understanding of how cellular identity and function are orchestrated. Regulatory elements (REMs) such as enhancers, repressors and promoters are non-coding DNA-regions regulating the expression of genes by serving as binding sites for Transcription Factors (TFs). Enhancers can also be transcribed to bi-directional enhancer RNA (eRNA) (1–4). REMs can be located far away from their target genes and affect them in an activating and/or a repressive manner (5–7).

Identifying REMs is difficult, as there is no method yet to locate them with absolute certainty. Instead, indirect epigenomic indicators are used in different combinations, leading to a variety of REM annotation approaches (8–11). Consequently, there are multiple publicly available REM platforms. The *Vista Enhancer Browser*, for example, contains tissue-specific REMs that were tested *in vivo* using transgenic mouse models. As the regulatory regions in the *Vista Enhancer Browser* database are experimentally validated, their number is limited (12). The *FANTOM5 Human Enhancers* website identifies REMs as part of the FANTOM5 project by analysing Cap Analysis of Gene Expression (CAGE) data to find eRNAs that show a bi-directional divergent transcription (13). *HACER* looks for eRNA as well, but additionally integrates GRO/PRO-seq (Global run-on sequencing/Precision Run-On Sequencing) data (14). While eRNAs are a clear indicator for the presence of a REM, they cannot pinpoint REMs that are not transcribed but act as TF binding sites (4).

Other REM resources include multiple different datasets. For instance, *GeneHancer* incorporates data from four different public REM databases and removes the redundant REMs (15). *RAEdb* interprets STARR-seq (self-transcribing active regulatory region-sequencing) and MPRA (Massively Parallel Reporter Assays) (16). *Enhancer Atlas 2.0* makes use of the broadest range of experimental methods with data from 12 techniques. With an unsupervised learning approach, it determines consensus REMs from the considered data types (17).

To understand the functionality of REMs, it is essential to know their target genes. Out of the websites named above, only *GeneHancer*, *HACER*, *FANTOM5 Human Enhancers* and *Enhancer Atlas 2.0* provide information on REM–gene interactions. Others offer the option to look for REMs in a defined window up- and downstream of a gene but do not presume any associations (12) or report REMs that overlap with promoter regions of genes (16). *GeneHancer* combines co-expression of eRNA, TFs, quantitative trait loci and chromosome conformation data to find target genes of REMs. *HACER* associates a REM to genes by integrating multiple chromosome conformation capture technologies (14). *FANTOM5 Human Enhancers* determines the pair-wise correlation between REM and gene expression (13). *Enhancer Atlas 2.0* uses the tool EAGLE to connect REMs to genes. EAGLE determines putative REM–gene interactions based on six different genomic features (17,18). Other platforms like *HEDD* (19) or *DiseaseEnhancer* (20) focus on the role of deregulated REMs in human diseases by examining their associations to disease-related genes.

EpiRegio uses the StitchIt algorithm which interprets chromatin accessibility data with respect to variation in gene expression (21). The algorithm identifies putative REMs that explain variation of gene expression across samples. In contrast to existing approaches, it starts with a gene and looks for REMs, not *viceversa*. It is important to note that REMs can overlap with each other. Consequently, REMs in close vicinity may act as coherent structures to regulate multiple different genes. To account for overlapping and adjacent REMs, we assign them to Cluster of REMs (CREMs) with a unique ID.

Further, StitchIt’s approach to interpret epigenomic variation in gene expression has the advantage of annotating regions that were observed in relation to actual gene expression changes, potentially leading to higher specificity. The REMs identified by StitchIt were shown to be better for predicting gene expression than REMs obtained from DNase1-seq peak calling. Different validation experiments further supported the validity of StitchIt’s REMs (21). In addition, StitchIt omits peak-calling as it is biased by the cut-off value or by variations induced by cell-cycle stages (22) and cell numbers (23). On top, EpiRegio quantifies a REM’s importance in a cell type-specific manner.

EpiRegio has convenient features aiding in the use of the website. Examples are available for every query to illustrate the input and possible options. All created tables can be downloaded in different formats. The EpiRegio REST API gives programmatic access to computational applications. EpiRegio is well documented and offers links to external websites like the Ensembl Genome Browser (24) or the UCSC Genome Browser (25) for further details.

## MATERIALS AND METHODS

### System setup

Our web server is developed with the Python-based web framework Django (v 2.2.10, Python 3.7). The result tables are created with the jQuery (v 1.19.1) library DataTables (v 1.10.20). The REST API is based on Django’s REST framework. Public access is provided by the Nginx (v 1.17.9) proxy service with Gunicorn (v 20.0.0) as the gateway interface. The source code is released under the GNU v3 license and is accessible at https://github.com/TeamRegio/EpiRegioDB. All necessary data behind our Epiregio web server is stored as a MySQL (v 8.0.19) database. We have also deposited a snapshot of the current version of our database in Zenodo https://doi.org/10.5281/zenodo.3750929 to ensure reproducible analyses using Epiregio and version control.

### Data processing

The data hosted by the web server was generated with StitchIt, an algorithm to identify REMs and simultaneously their target genes by interpreting epigenetic signal variation in relation to changes in gene expression. StitchIt was applied to human paired DNase1-seq and RNA-seq data, namely 110 samples from the Roadmap consortium (26) and 56 samples from the Blueprint consortium (27). The considered samples comprise of 46 different tissues and cell types. While the Blueprint dataset consists of various primary cell types and disease related samples associated to the haematopoietic system, Roadmap data provides a broader diversity of cell and tissue types. All datasets have been uniformly preprocessed. DNase1-seq was adjusted to sequencing depth and gene expression is quantified in transcripts per million.

For every gene, StitchIt inspects a user-defined region around the gene to determine putative associated REMs. For the data provided in EpiRegio, we consider a window of 100 000 bp upstream of a gene’s transcription start site, the entire gene body and the window of 100 000 bp downstream of a gene’s transcription termination site. Hence, even distant REMs are taken into account. A two-level machine learning approach is used to learn the associations of REMs to a gene. After a linear regression using elastic-net penalty for feature selection, an Ordinary Least Squares (OLS) regression model determines a final regression coefficient and its corresponding *P*-value. The *P*-value assesses the contribution of an individual REM to the predictability of a gene’s expression across the considered tissues and cell types. For a detailed explanation of the computational method of StitchIt see (21).

EpiRegio contains a total of 2 404 861 REMs associated to 35 379 protein-coding and non-protein coding genes. The average length of a REM is 229 bp (±235 bp). For each REM, scores based on the regression coefficient and the corresponding *P*-value obtained from the OLS model are reported (for more detailed information see ‘Output’ section).

Different REMs can overlap with each other. To allow for the analysis of these REMs we introduce Cluster of Regulatory EleMents (CREMs). Each CREM is formed by REMs that overlap (by at least 1 bp) or are adjacent (0 bp in between two REMs) to each other and contains a minimum of two REMs. As the REMs of a CREM are known, it is possible to derive the regulatory potential of each part of the CREM. Approximately half of all REMs are part of a CREM. Together, they form 365 286 distinct CREMs, which contain 3.5 REMs on average and span 534 bp (±460 bp), see Table 1. There are 9786 CREMs in which all contained REMs overlap completely with each other.

**Table 1.** Quantitative characteristics of REMs and CREMs

	Mean	Std	Min	Max
REM length [bp]	228.9	234.7	4	1999
CREM length [bp]	533.6	460.0	11	8752
REMs per CREM	3.5	2.9	2	122
Associations to different genes	2.8	1.4	2	31

REMs per CREM refers to the number of REMs that form a CREM. Associations to different genes shows to how many different genes the REMs inside of a CREM are linked to.

Further, EpiRegio enables the comparison of REM activity across different tissues and cell types based on DNase1-seq data.

### Input

**Figure 1.**
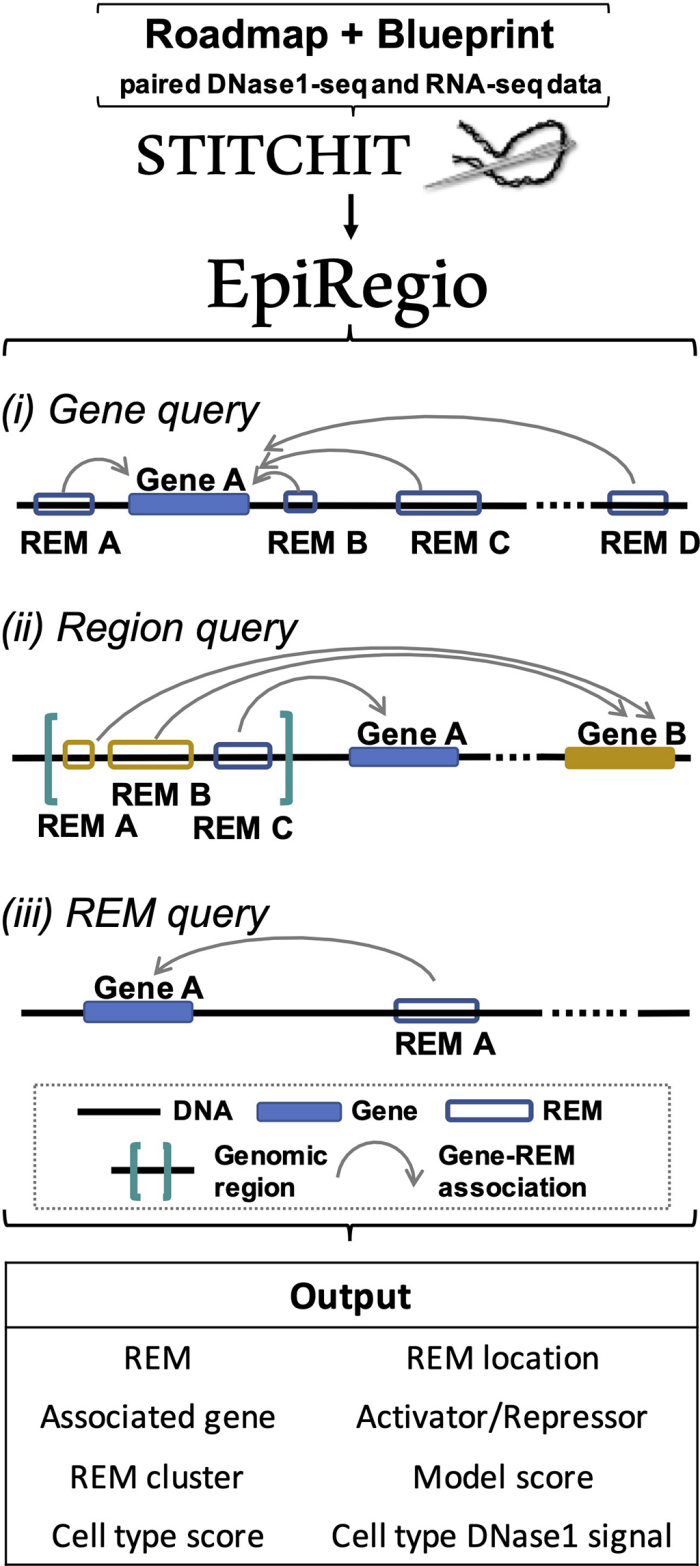
Structure of EpiRegio. Human paired DNase1-seq and RNA-seq data of the Roadmap and Blueprint consortium were used to annotate REMs and their target genes with StitchIt. EpiRegio allows for three types of queries: users can either search for REMs associated to their genes of interest, look for REMs in genomic regions or query REMs by their ID directly. Every query results in an interactive table containing all the parameter shown under Output.

The EpiRegio web server allows three types of queries (see Figure 1).

#### Gene query

Users can query for genes of interest by providing Ensembl IDs (GRCh38.p10) or gene symbols as input. All REMs that link to the inspected genes will be presented as output.

#### Region query

Another option is to use genomic regions as input to receive information on all overlapping REMs. The amount of overlap can be selected by the user. By default only REMs that overlap entirely with the targeted regions will be returned.

#### REM query

The last query type accepts REM IDs as input, in order to investigate REMs of interest directly. It is meant for users who are already familiar with our nomenclature of REMs.

Each query provides the option to upload a csv- or txt-file as input (e.g. a list of gene symbols) for a more efficient workflow. The query for regions in Figure 1(ii) also allows upload of files in BED-format. We provide example files for each query in the Supplementary Material. Every query can be further specified by a selection of tissue and cell types to compute cell type-specific parameters, such as the DNase1 signal. It is possible to set a threshold for the *Cell type DNase1 signals* that restricts the query to REMs that exceed this threshold in all of the selected cell types or tissues. In addition, EpiRegio includes a REST API based on Django’s REST framework for more systematic accessing options and the possibility to be included in automated computational processes. It can either be used in the browser or via a program that is capable of making HTTPS requests. An example for an easy-to-use tool to build such a program is the Python package *requests*. A detailed guide for each of the queries, as well as for the use of the REST API is available in our documentation (https://epiregiodb.readthedocs.io/en/latest/).

### Output

**Figure 2.**
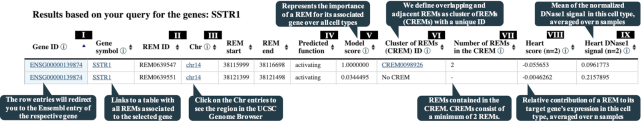
First two rows of an example result table of EpiRegio’s *Gene query* for SSTR1.

The three main query types result in an interactive table showing the REMs that match the user’s query settings. See Figure 2 for an example output table of a *Gene query*. In the following sections, the information provided per REM will be explained in detail.

#### Gene ID (I), REMID (II), Genomic location (III)

Every row represents one REM with its ID, its associated gene (both ensembl ID and gene symbol) and the REM’s genomic location, specified by the chromosome, as well as the start and end position.

#### Predicted function (IV)

The column *Predicted function* displays whether a REM was associated with an activation or repression of its gene. It is based on the sign of the regression coefficient. A regression coefficient above zero indicates an activating effect. Hence, a negative coefficient implies a repressive function of the REM.

#### Model score (V)

The *Model score* is the normalized absolute binary logarithm of the *P*-value (range [0, 1]) obtained by testing the importance of a REM for the expression prediction of its target gene. The closer the score is to 1, the higher the predicted impact of the REM for its target gene. This value is not cell type-specific, meaning that in some cell types a REM with a high *Model score* can potentially be less important than another one with a lower score.

#### Cluster of REMs (CREM) ID (VI) and Number of REMs in the CREM (VII)

If two or more REMs overlap or are adjacent to each other, we assign them to a CREM with a unique ID. *Number of REMs* shows the number of REMs contained in a particular CREM. The column is empty if a REM has no adjacent or overlapping REM. By clicking on a *CREM ID* the user gets redirected to a table with all REMs in the cluster.

#### Cell type score (VIII)

In order to study the potential contribution of a REM to its target gene in a given cell type, we introduce the *Cell type score*. This score denotes a normalized quantity in [ − 1, 1] that estimates the relative contribution to the gene’s expression (positively or negatively) by the REM *r* in cell type *c*:

(1) $$\begin{equation*} \begin{split} {\rm Cell\,type\,score}(r,c):=\frac{\beta _{r}\cdot DNase1\text{-}{\rm signal}_{r,c}}{\sum \limits _{r_{i}\in R}\,\,|\beta _{r_{i}}\cdot DNase1\text{-}{\rm signal}_{r_{i},c}|}. \end{split} \end{equation*}$$

The regression coefficient (β) describes the association between a REM and its gene’s expression. The DNase1-signal is log-transformed and standardized for each REM over all cell types (mean = 0, standard deviation = 1) and represents how active a REM is in a cell type *c*. *R* is defined as the set of all REMs associated to a given gene, thus *R* = {*r*_1_, …, *r*_*n*_}. The *Cell type score* normalizes the contribution of REM *r* to its gene’s expression in this specific cell type as predicted by the linear model. As there are sometimes multiple samples per cell type the *Cell type score* is averaged over all samples. The regression coefficient is not cell type-specific, but determined per REM-gene association. If multiple cell types are selected for one query, the score will be calculated for each cell type separately, independent of the other selected cell types. The *Cell type score* can be used to rank the REMs according to their importance between cell types for the same gene or to compare the importance of different REMs within a cell type (see ‘Application Scenarios’ section).

#### Cell type DNase1 signal (IX)

The *Cell type DNase1 signal* is defined as *log*_2_(*DNase*1 *signal*). It serves as a measure of how accessible the chromatin is in the REMs and indicates the activity of a REM. It is retrieved from the Roadmap and Blueprint datasets. As mentioned beforehand, we have multiple samples for each cell type. The signal is averaged over all available samples of a cell type. When performing a query with multiple cell types, the *Cell type DNase1 signal* will be determined for each cell type separately. Since the DNase1 signal is normalized for sequencing depth, it allows for the comparison of chromatin accessibility between samples.

All result tables can be downloaded as excel- or csv-file.

## APPLICATION SCENARIOS

In this section we illustrate how EpiRegio can be used to conduct more advanced analyses: We present two example application scenarios, based on information obtained from our web server.

### Elucidation of disease pathways directly from a TF-ChIP experiment

A common question in the analysis of TF-ChIP-seq data is to identify the TF’s target genes using ChIP-seq peak regions. Simple association approaches, like using a window around gene start sites or associating a peak to the nearest gene, are often inaccurate (28). Here, we illustrate how to use TF-ChIP-seq binding regions to learn about the biological function of TF target genes.

We downloaded the binding locations of the TF ARID3A from the ENCODE database (Accession: ENCFF002CVL) as a BED file which contained 9026 TF-ChIP peaks. We searched for REMs overlapping at least by $50\%$ with the TF-ChIP peaks using the *Region query* in the EpiRegio webserver. The resulting REMs were associated with 1721 unique genes. We subjected them to a functional enrichment analysis using g:Profiler (29) with default parameters, except setting the significance threshold to 0.05 using the Benjamini–Hochberg FDR method. The g:Profiler analysis can be reproduced using the following link: https://biit.cs.ut.ee/gplink/l/3i6bF7IGRS.

There were two KEGG metabolic pathways enriched in the analysis: the systemic lupus erythematosus (SLE) disease pathway and genes associated with alcoholism. SLE is an autoimmune disease affecting multiple organs, whose cause is unknown till date. Interestingly, the disease severity of SLE has been shown to increase with the expression of ARID3A (30). Also, ARID3A has been identified in the hypermethylated network of genes in an association study of alcohol use disorder (31). As we have used different data, our experiment constitutes another independent association of ARID3A and alcohol consumption.

Further, we identified *chromatin assembly or disassembly* and *nucleosome organization* as the top two enriched biological processes among ARID3A target genes. While it is unsurprising to see a TF being involved in chromatin regulation, the ARID family of genes was specifically shown to be involved in chromatin regulatory complexes (32). These results exemplify how researchers can elucidate disease pathways with minimal downstream analyses of the output from our server’s *Region query* search.

### Identify enriched transcription factors of differentially expressed genes

In this application scenario, we show how EpiRegio can be used to explore which TF binding sites are enriched in REMs of genes of interest. The aim is to identify key TFs involved in functional mechanisms or gene regulation pathways of the analysed genes.

The following analysis is based on a single-cell RNA-seq dataset from Glaser *et al.* (33), where Human Umbilical Endothelial Cells (HUVECs) were treated with TGF-β to trigger an endothelial-to-mesenchymal transition (EndoMT). For this application, we analysed genes that are differentially expressed (up- or down regulated) in the TGF-β-treated cells in comparison to untreated HUVECs. To compute the differentially expressed genes, we used Seurat’s FindAllMarkers function, which performs a Wilcoxon Rank Sum test (*P*-value ≤ 0.01). We identified 11 836 corresponding REMs for 304 differentially expressed genes (see Supplementary Material) using EpiRegio’s gene query functionality.

Next, we applied PASTAA (34) as TF motif enrichment tool, which requires the DNA sequences of the REMs and a set of known TF binding motifs as input. We determined the DNA sequences with bedtools (35) and downloaded 515 TF binding motifs from the JASPAR database (36). In addition, PASTAA asks for a ranking of regions. We sorted the REMs based on the *Cell type score*. We chose *heart* as tissue, as $\approx 10\%$ of the endothelial cells within the heart undergo EndoMT during cardiac development, as well as $\approx 1\%$ in the adult heart. Further, EndoMT takes place during myocardial infarction (37).

We adjusted the resulting *P*-values from PASTAA using the Benjamini–Hochberg FDR procedure. We considered a motif of a TF as enriched with an adjusted *P*-value ≤ 0.05, which resulted in 230 different TFs (see Supplementary Material). Within this result, we found several TFs commonly known to play a crucial role in EndoMT, e.g. *SP1*, *NFKB1* (38,39), as well as Smad transcriptional regulators like *SMAD3* (40).

Overall, this analysis illustrates how researchers can use EpiRegio’s *Gene query* with a commonly used downstream analysis to infer key TFs for various phenotypes and conditions. This is a first step to unravel possible regulatory pathways of a gene set of interest.

## DISCUSSION

EpiRegio is unique with its approach to identify REMs. It starts with a gene and looks for putative REMs that could induce variation in gene expression across different samples. This is different to related methods that first define a REM by using CAGE or epigenomics data and then use varying approaches for associating the REMs with genes, e.g. (13–15). By using EpiRegio, we can avoid simplistic methods like linking a REM to its nearest gene.

In our first application scenario, we analysed ChIP-seq data of the TF ARID3A to explore the functionality of genes that show associations to the binding regions of ARID3A. We were able to recapture findings from previous studies by showing that ARID3A is involved in regulating a network of genes found in pathways of SLE and alcoholism. In summary, combining EpiRegio with standard analysis tools allows us to identify cellular pathways that are affected by TFs of interest.

In a second application, we demonstrate how EpiRegio can be used to identify key TFs enriched in REMs of differentially expressed genes in proliferating cells. For this analysis we decided to use a TF motif enrichment tool which additionally requires cell type-specific information. Namely, we used PASTAA, which identifies TF binding motifs that are significantly enriched in high ranked input sequences compared to low ranked sequences. In order to rank the obtained REMs we sorted them according to their *Cell type score*. There is a plethora of different TF motif enrichment tools available and each of them is suitable for different tasks. Some tools identify enriched TF motifs based on a set of DNA regions provided by the user e.g. Clover (41), MotifCounter (42) or Homer (43). Tools such as i-cisTarget (44) additionally incorporate publicly available epigenomics data to identify enriched motifs in user-provided DNA regions. Therefore, EpiRegio provides the user with the flexibility to decide which TF motif enrichment tool is the most suitable for the analysis of interest.

We would like to mention that the linear model used in STITCHIT, which forms the basis for assessing the *Cell type score*, is a simplification to the actual regulation of genes by their REMs. The linear model considers the contribution of each REM to be independent and additive, which is unlikely to be true for all REMs and currently debated in the field (45). We hope that through the use of these scores, we can identify genes, where this approximation is helpful and otherwise investigate the use of more sophisticated scoring schemes.

## CONCLUSION AND FUTURE DIRECTIONS

With EpiRegio we built an easy-to-access tool to efficiently retrieve regulatory regions and their associated genes. In our presented application scenarios we showed that EpiRegio can be used for a range of different datasets and can be included in various kinds of analyses.

The different interfaces of our web server are intuitive to use and allow for various kinds of queries. Our REST API enables users to access data programmatically. Extensive unit testing ensures a stable functionality of the server. EpiRegio will be further refined and expanded. As consortia like the International Human Epigenome Consortium are continuously making more datasets available, we are planning to include more species beside of human, as well as more cell and tissue types. More functionalities will be added to EpiRegio to provide users a broader range of tools and epigenome analyses.

We believe that EpiRegio is a valuable tool in unravelling the complex network of gene regulation. It can be the basis for a variety of scientific questions and represents a source of information that is relevant in many different scenarios, like understanding the regulatory network of one or multiple genes, finding target regions for experimental setups or looking into putative target genes of TFs.

## Supplementary Material

gkaa382_Supplemental_Files
